# Landscape effects on the thermotolerance of carabid beetles and the role of behavioral thermoregulation

**DOI:** 10.1111/1744-7917.13044

**Published:** 2022-05-16

**Authors:** Lucy Alford, Sacha Roudine, Jean‐Sébastien Pierre, Françoise Burel, Joan van Baaren

**Affiliations:** ^1^ UMR 6553 ECOBIO, Université de Rennes I Rennes Cedex France; ^2^ School of Biological Sciences University of Bristol Bristol UK

**Keywords:** agroecosystems, local adaptation, physiological thermal tolerance, plasticity, winter resistance

## Abstract

Physiological thermotolerance and behavioral thermoregulation are central to seasonal cold adaptation in ectothermic organisms. For species with enhanced mobility, behavioral responses may be of greater importance in the cold stress response. Employing the carabid beetles as a study organism, the current study compared physiological thermotolerance and behavioral thermoregulation in carabid species inhabiting cereal fields in different landscape contexts, from fine grain heterogeneous “complex” landscapes to homogenous “simple” landscapes. Physiological thermotolerance was determined via measurement of the CT_min_ and chill coma temperature. Behavioral responses to cold temperature exposure were determined employing a purpose built arena, and thoracic temperature measured to estimate the efficacy of the behavior as a form of behavioral thermoregulation. Results revealed an influence of landscape composition on the cold tolerance of carabid beetles, although species differed in their sensitivity to landscape intensification. A reduced effect of landscape on the thermotolerance of larger carabid beetles was observed, thought to be the consequence of greater mobility preventing local acclimation to microclimatic variation along the landscape intensification gradient. Investigation into behavioral thermoregulation of the 3 largest species revealed burrowing behavior to be the main behavioral response to cold stress, acting to significantly raise carabid body temperature. This finding highlights the importance of behavioral thermoregulation as a strategy to evade cold stress. The use of behavioral thermoregulation may negate the need to invest in physiological thermotolerance, further offering explanation for the lack of landscape effect on the physiological thermotolerance of larger carabids.

## Graphical abstract

Physiological thermotolerance and behavioral thermoregulation was studied in carabid species inhabiting cereal fields of different landscape complexities. A reduced effect of landscape on the thermal tolerance of larger carabid beetles was observed, thought to be the consequence of greater mobility preventing local acclimation to microclimatic variation. Burrowing behavior was found to be the main behavioral response to cold stress. This finding highlights the importance of behavioral thermoregulation as a strategy to evade cold stress. The use of behavioral thermoregulation may negate the need to invest in physiological thermotolerance.

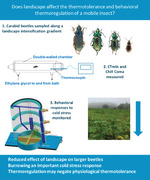

## Introduction

When faced with predictable unfavorable cold temperatures associated with temperate winters, a range of coping strategies are available to ectothermic organisms to enable them to persist in a thermally stressful environment. Firstly, the individual may avoid exposure to the unfavorable conditions altogether and migrate to a more suitable climate (Larsen & Lee, 1994). Alternatively, the individual may remain in the thermally stressful environmental and, in doing so, must either enter into diapause, a genetically determined state of decreased metabolic activity and developmental arrest (Denlinger, 2002; Teets & Denlinger, 2013), or instead maintain activity. Central to the ability to remain and endure the challenges associated with temperate winters is the capacity to respond to environmental cues such as shortening day lengths and declining temperatures to trigger seasonal cold adaptation (Teets & Denlinger, 2013). Such seasonal adaptation is incurred via adaptive phenotypic plasticity and may encompass short‐term changes to the physiology or behavior of the individual that act to enhance thermotolerance (May, 1979; Overgaard & MacMillan, 2017), to longer‐term transgenerational plasticity (Massamba‐N'Siala *et al*., 2014; Walsh *et al*., 2014). In ectothermic organisms, physiological changes to enhance thermotolerance may include changes to homeostatic balance, lipid membrane composition, cryoprotective osmolytes, and heat shock protein expression (Overgaard & MacMillan, 2017). In addition to physiological plasticity, phenotypic plasticity may also encompass behavioral alterations which further act to enhance thermotolerance and assist in seasonal cold adaptation. Indeed, behavioral changes such as alterations to foraging behavior (Andrew *et al*., 2013), overwintering strategy (Tougeron *et al*., 2017), choice of microhabitat (Alford *et al*., 2017), and level of sociality (Schürch *et al*., 2016) may offset the effects of changing temperatures.

Studies into the plasticity of thermotolerance are commonly performed at the local scale (*e.g*., Powell & Bale, 2005, 2006; Sinclair & Chown, 2006; Andrew *et al*., 2011; Renault *et al*., 2012). However, with landscape components such as topography, vegetation type and cover known to directly impact the microclimate (Chen *et al*., 1999), studies have more recently examined physiological thermotolerance at the landscape scale to elucidate how landscape composition can impact the acclimatory response of ectotherms (Tougeron *et al*., 2016; Alford *et al*., 2018). Indeed, simplified landscapes of intensive farming regimes display significantly different temperature means and extremes to wooded landscapes, with species reacting in accordance to their respective sensitivity to mean and extreme temperatures (Alford *et al*., 2018). Furthermore, plasticity in physiological thermotolerance, although adaptive in winter when individuals are exposed to cold stress, becomes markedly reduced in spring, with any effect of landscape being reduced or lost completely (Tougeron *et al*., 2016; Alford *et al*., 2018). Ectothermic species may thus respond to microclimatic variation brought about by variation in landscape composition, and alter seasonal acclimation accordingly. In 2 related studies into the effect of landscape complexity on insect physiological thermotolerance, it was found that the cereal aphids *Metopolophium dirhodum*, *Rhopalosiphum padi*, and *Sitobion avenae* (Hemiptera: Aphididae) and their parasitoid wasps *Aphidius avenae*, *A. ervi*, *A. matricariae*, *A. rhopalosiphi* (Hymenoptera: Braconidae) differed in their response. Here, aphids from more “complex” farming landscapes (landscapes which retained a high proportion of semi‐natural features such as hedgerows and grasslands) displayed an enhanced cold tolerance compared to their counterparts from intensively farmed “simple” landscapes (Alford *et al*., 2018). The reverse was true of aphid parasitoids, with parasitoids from intensively farmed landscapes displaying the greatest cold tolerance (Tougeron *et al*., 2016), indicating that insects may not respond in a similar manner to landscape change. Cereal aphids and parasitoid wasps are comparatively small insects (Alford *et al*., 2018) dependent upon a host plant and host aphid, respectively, and thus with limited mobility. This is particularly true of aphids in their apterous form which predominate in the study area. How larger insect species with enhanced mobility respond to landscape composition is currently unknown. However, any ability to utilize behavioral thermoregulation to buffer thermal variation may see a reduced plasticity in physiological thermotolerance and, as such, any landscape effect on physiological thermotolerance may be reduced or lacking completely.

Employing the ground beetles (Coleoptera: Carabidae) *Agonum muelleri*, *Amara aenea*, *Anchomenus dorsalis*, *Bembidion lampros*, *Bembidion quadrimaculatum*, *Bembidion tetracolum*, *Harpalus rufipes*, *Poecilus cupreus*, *Pterostichus melanarius*, and *Trechus quadristriatus* as a study group, the current study aimed to investigate landscape effects on physiological thermotolerance and the role of behavioral thermoregulation in the cold stress response of a group of insects considered highly mobile at the local scale (Wallin & Ekbom, 1988; Jopp & Reuter, 2005). With over 40 000 species (Lövei & Sunderland, 1996), the carabids represent a diverse and cosmopolitan family of insects, exhibiting great diversity in traits including size (Jelaska & Durbešić, 2009), breeding season (Den Boer & Den Boer‐Daanje, 1990), and diet (Sunderland, 1975; Jelaska *et al*., 2014) to name a few. Many species of carabids are generalist predators and opportunistic feeders, capable of altering the relative consumption of prey species to match their seasonal abundance (Sunderland, 1975; Chiverton, 1987; Wheater, 1989; King *et al*., 2010; Jelaska *et al*., 2014). For this reason, carabids play an important role in the biological control of herbivorous pests in temperate agroecosystems (Collins *et al*., 2002; Sunderland, 2002; Al Hassan *et al*., 2013). During winter months, carabids commonly overwinter within the soil in a state of diapause (Holland *et al*., 2007). However, recent evidence suggests that carabids are becoming increasingly winter active in temperate agricultural landscapes as a consequence of warming temperatures (Damien, 2018). This increase in winter activity renders carabids increasingly susceptible to the cold stresses imposed by a temperate winter and places increased importance on physiological and behavioral thermotolerance to withstand unfavorable cold temperatures. Using cereal fields (maize, wheat) of North‐West France as a study system, we report on a 2 year study, comprising 4 biannual sampling seasons, investigating landscape effects on carabid beetle physiological thermotolerance along a landscape complexity gradient and the role of behavioral thermoregulation in the cold stress response. By employing the same ecosystem, region, and landscape gradient as previously utilized by Tougeron *et al*. (2016) and Alford *et al*. (2018), results obtained for carabid beetles in the current study will be directly comparable to responses previously observed for the insects of other studied families. The following hypotheses were tested: (1) Due to complex landscapes having lower mean temperatures than simple landscapes, it is hypothesized that carabid physiological thermotolerance will significantly differ with landscape complexity with carabids originating from complex landscapes being the most cold tolerant. (2) Exposure to unfavorable cold temperatures will be less frequent in spring months compared to autumn months, eliciting a lower phenotypic response in carabid thermotolerance. Landscape effects on carabid thermotolerance will therefore be more pronounced in autumn and less pronounced or absent in spring. (3) There will be a reduced effect of landscape on the thermotolerance of larger carabids as a result of enhanced mobility to move through the landscape and evade unfavorable thermal conditions. (4) Carabid beetles will display behavioral thermoregulation to escape unfavorable cold temperatures. Active engagement in behavioral thermoregulation will enable the carabid to alter its core body temperature compared to carabids unable to engage in behavioral thermoregulation. (5) More cold tolerant carabid species will be less reliant on behavioral thermoregulation than less cold tolerant carabids.

## Materials and methods

### Carabid collection and storage

The current study utilized a landscape gradient previously established as part of the long‐running project “FARMLAND” (farmland‐biodiversity.org) in the Long‐Term Ecological Research (LTER) site ZA Armorique (48°36′N, 1°32′W) (https://osur.univ‐rennes1.fr/za‐armorique/) in the Brittany region of France. Here, complex farming landscapes are classified as fine grain heterogeneous landscapes characterized by high diversity, high hedgerow density (>3 200 m), small field sizes (<0.93 ha), and the presence of grassland areas (>45%). In contrast, intensified farming landscapes are classified as more homogenous landscapes characterized by low diversity, large field sizes (>2.70 ha), and few seminatural elements (grassland density <20% and low hedgerow density <550 m) (Tougeron *et al*., 2016; Alford *et al*., 2018). Utilizing this preexisting landscape gradient, carabid beetles were sampled over 4 consecutive sampling seasons: autumn 2013 (commencing 9th September), spring 2014 (commencing 24th March), autumn 2014 (commencing 3rd September), and spring 2015 (commencing 30th March) for a duration of 4–5 weeks. In each of the 4 sampling seasons, a total of 9 cereal fields from conventional, nonorganic farms were selected along the landscape intensification gradient, with 3 fields selected to represent a “complex” landscape, 3 fields an intensified “simple” landscape, and 3 fields an “intermediate” landscape. Due to sampling in agricultural landscapes, the fields selected for sampling were dictated by the crop rotation regime of the farmer. As a consequence, sampling fields varied season on season. During each sampling season, a total of 8 pitfall traps were dug in each field for the collection of carabids, spaced at a distance of 1 m apart and 5 m from the field margin. Traps comprised a plastic cup measuring 85 mm in diameter and 110 mm in depth. Traps were dug to a depth so that the entrance of the trap was flush with ground level. A “roof,” comprising a piece of Perspex measuring approximately 150 mm × 100 mm attached to a plastic stick, was positioned over each trap to prevent the trap filling with rainwater. A slice of apple and 3–4 pieces of dry dog food (Brekkies Excel, Multicroc by Affinity Petcare) were placed within each trap to provide a food source for captured carabids until the traps were emptied. Trap contents were collected every other day, approximately twice per week on a Wednesday and Friday, for the 4–5 weeks duration of the sampling season. At the end of a sampling week on the Friday, traps were closed with a plastic lid and reopened the following week on the Monday to prevent carabids being trapped in the pitfall traps for extended periods of time over the weekends.

Collected adult carabids were returned to the laboratory at the Université de Rennes 1. Individual Perspex boxes were created to house the carabids and measured 175 mm × 115 mm × 65 mm with a ventilated lid. Boxes were lined with a layer of compost, approximately 20 mm deep, and roughly 1 quarter of the surface area was covered with moss. Carabids were crudely sorted into species and placed within boxes at densities of approximately 20 per box to reduce the possibility of cannibalism. All carabids were fed twice weekly on sliced apple and dry dog food (Brekkies Excel, Multicroc by Affinity Petcare) and the boxes misted with water. All boxes were housed in a temperature‐controlled climate chamber at 20 ± 1°C and LD 16 : 8 h for the minimum duration until use in experiments.

### Cold tolerance measurements

Two indices of cold tolerance were determined for carabid beetles collected in the field. These thresholds included (1) the temperature at which spontaneous movement ceases and the insect is rendered immobile (referred to here as the Critical Thermal Minima or CT_min_); and (2) the temperature at which the insect enters a cold induced torpor (chill coma), commonly defined by the last twitch of an appendage. These 2 thresholds refer to stages 2 and 4 in Hazell and Bale's 4 stages of the chill coma process (Hazell & Bale, 2011).

Critical thermal minima and chill coma thresholds were measured using a glass column, as described in Powell and Bale (2006). The glass column (35 × 5 cm), similar in design to a condenser column, was connected to a programmable alcohol bath (Haake F3, Thermo Electron Corp., Karlsruhe, Baden‐Württemberg, Germany), enabling the circulation of ethylene glycol around the outer chamber and thus fine control over the air temperature experienced within the inner column. In all experiments, the glass column was positioned horizontally and preset to the culture temperature of 20°C. Carabid beetles were inserted into the column at densities of 2–3 individuals (depending on size) and the column was subsequently closed with a sponge stopper to reduce air flow and maintain a stable thermal environment within the inner column. Densities of 2–3 carabids per test run enabled a more rapid test rate, whilst ensuring minimal interaction during threshold measurement. On the rare occasion that test carabids grouped together during threshold measurement, these data were excluded and the experiment was restarted with fresh carabids. Following a 10 min acclimatization period, the programmable alcohol bath was set to decrease the temperature of the column from 20°C to −10°C at a rate of 0.5°C/min. The cooling rate was chosen to prevent the rapid cold hardening of test insects (Powell & Bale, 2006). During the cooling phase, the temperature of CT_min_ was determined for each carabid. Chill coma was determined for the larger species of carabid only (*H. rufipes*, *P. cupreus*, and *P. melanarius*) since the larger size facilitated detection of the last twitch of an appendage. Critical thermal minima and coma threshold temperatures were recorded manually using a type T thermocouple probe (VWR, Fontenay‐sous‐Bois, France), accurate to 0.01°C, connected to a digital display.

Following completion of the experiment, each carabid (now in a state of chill coma) was removed from the glass tube and placed individually in an Eppendorf tube containing 70% ethanol solution for preservation. The experimental procedure was repeated to obtain CT_min_ and coma threshold temperatures for all carabids collected during a sampling season, which equated to approximately 450–500 carabids per season. This resulted in data for 1962 carabids over the 2 years. Species identification was performed for all carabids under a stereomicroscope using the identification key of Roger *et al*. (2012).

### Carabid size

Following determination of cold tolerance traits, carabid size was subsequently determined by measuring the distance between the labrum and the posterior end of the elytra (Roger *et al*., 2012). Measurements were made using a camera (Zeiss AxioCam ERc5s^®^ HD) mounted on an ×9 binocular microscope. Photographs were taken using Intelcam software and morphological measurements made using the image processing software Image J^®^ (v. 1.48). Size was determined for a total of 948 carabids over the 2‐year sampling period and the effect of size on carabid thermotolerance was investigated using this subset. The size of *B. quadrimaculatum* was not determined due to a lack of individuals when performing the size calculations.

### Behavioral thermoregulation

**Fig. 1:**
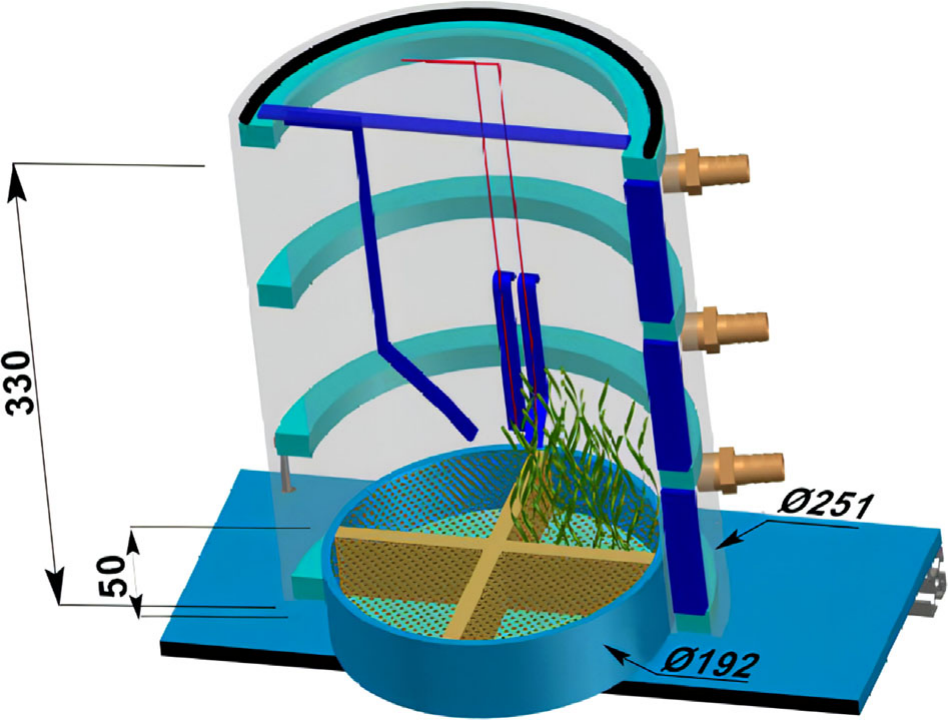
Observation arena developed to study carabid behavioral thermoregulation. The doubled‐walled nature of the arena, in conjunction with a secondary circuit built into the base, enabled the circulation of ethylene glycol from a programmable alcohol bath around the cylinder to control the temperature experienced within the arena. The floor of the arena was divided into 4 different substrates: (1) wheat grown in potting compost at a density of 15 blades of wheat and approximately 15 cm in height; (2) bare potting compost; (3) bare potting compost in which 3 holes of 1 cm in width and depth were drilled; and (4) potting compost with an artificial shelter. Dimensions are provided in mm.

Behavioral thermoregulation was monitored in a subset of carabids comprising the 3 largest species collected during the spring 2015 sampling season from the intermediate landscape: *H. rufipes*, *P. cupreus*, and *P. melanarius*. The largest species were chosen for ease of behavioral observations. All carabids were returned to the laboratory and stored as detailed above. A purpose‐built observation chamber was designed that comprised 2 cylinders of Plexiglas which could be connected to a programmable alcohol bath. The doubled‐walled nature of the arena, in conjunction with a secondary circuit built into the base, enabled the circulation of a cooling fluid (ethylene glycol) around the entirety of the cylinder with the exception of the open top of the cylinder which was eventually closed with a Plexiglas lid and rubber seal (Fig. 1). The base of the arena was divided into 4 sections, enabling the recreation of 4 artificial habitats: (1) wheat grown in potting compost at a density of 15 blades of wheat per section and approximately 15 cm in height; (2) bare potting compost; (3) bare potting compost in which 3 holes of 1 cm in width and depth had been drilled; and (4) potting compost with an artificial shelter made of thin cardboard at a height of 3 cm. An artificial shelter was used to crudely represent leaf litter since use of leaf litter obstructed the carabid from view and interfered with behavioral observations.

For all experiments, the observation arena was housed in a temperature‐controlled room held at 15 ± 1°C under homogeneous white light, 250 lux. Prior to the experiment, an individual carabid was placed in the arena and allowed to acclimate to their new environment for a duration of 30 min. After this initial acclimation period, the programmable alcohol bath was programmed to decrease the temperature from the starting temperature of 15–0°C at a rate of 0.25°C/min to produce an experimental duration of 1 h. A thermocouple was placed in the center of the arena to record the air temperature within the arena. During the cooling phase, carabid behavior was recorded every 0.5°C and the following behaviors were scored: walking, running, burying, grooming, or inactive. Following completion of the temperature ramp, the carabid was removed and the body temperature of the beetle was immediately measured by inserting a thermocouple probe into the thorax of the carabid. A temperature control group was set up in which the carabids were not subjected to the declining temperature regime, but instead remained at the starting temperature of 15°C.

To test whether the ability to actively interact with the environment enabled the carabid to engage in behavioral thermoregulation and alter its body temperature, a second behavioral control was performed in which the bottom of the arena was filled with a layer of potting compost covered by a nylon mesh. The arena contained no features which enabled the carabid to climb or shelter and the nylon mesh acted to prevent the carabid from burrowing. For the behavioral control, a carabid was placed in the arena and the temperature was decreased as detailed above. On reaching 0°C, the thoracic temperature of the carabid was measured as above.

### Statistical analysis

Since CT_min_ is fundamentally a delay, CT_min_ data were analyzed using a Cox proportional hazard model which belongs to the class of generalized linear models (GLMs) typically applied to survival data which are not normally distributed (exponential, Weibull, Gamma, *etc*.) (Alford *et al*., 2018). Of the 31 and 38 carabid species collected in the autumn and spring sampling period, respectively, 10 species were present across the 3 landscape types with at least 10 individuals for each type. A Cox proportional hazard model was subsequently built with data from the 10 species to determine any effect of landscape type and sampling season on the cold tolerance (measured by CT_min_) of carabid beetles using the *coxph* function of the “*survival*” package of R software (R Core Team, 2019). Here, we take advantage of the confusion between time and temperature to treat the CT_min_ as a failure time. Cox models give access to all classic models of variance (deviance) analysis, covariance, and regression. The Cox model can be interpreted as the failure rate at time *t* and can be estimated as follows:

$$\lambda \left(t\right)=\lambda_{0}\left(t\right)\exp\sum_{i=1}^{p}\beta \mathrm{iXi},$$

where *λ*_0_ is the baseline hazard rate that corresponds to the value of the hazard rate if all the covariates are equal to zero and βi is the regression coefficients that measure the impact of the covariates. A stepwise model selection was used based on the Akaike information criterion (AIC) to estimate predictor importance. Each field was considered as a frailty factor (random factor) and the significance of the interaction between the species and landscape type was tested. The overall model tested was the following: Surv (CT_min_) = intercept + species × landscape + sampling season + frailty(field) + error. Analysis of deviance tests are issued from the *χ* ^2^ approximation of the deviance likelihood ratio. As the interaction was significant in the selected model, the crossed levels species × landscape type was studied. The “survfit” function was used to calculate estimates of the different “survival” curves (hereafter referred to as failure curves since the current study applies the method to cold tolerance data) using the Kaplan–Meier estimators. Pairwise differences between failure curves were studied using the log‐rank test with the function *pairwise_survdiff* of the package *survminer* (Kassambara *et al*., 2021), adjusting *P*‐value with the Benjamini and Hochberg's method (Benjamini & Hochberg, 1995).

Looking at carabid cold tolerance, a high interaction between landscape type and carabid species was found. As a consequence, landscape types were considered as strata to test inter‐species differences in cold tolerance. As the number of carabid beetles was unbalanced between landscape type and in order to compare stratified samples of several species, the method of moments estimators was used. An unbiassed estimator of the mean CT_min_ for each species was independently built on the basis of the combination of a clustered sampling (fields) nested within a stratified sampling (landscape type). The details of the calculus are given in Appendix 1. CT_min_ of all species were then compared 2 by 2 with a Bonferroni post hoc procedure.

To assess the effect of cold exposure on the behavior of carabids, as well as behavioral differences between the 3 species, GLM with a Poisson distribution error were built. Separate GLM were performed for each behavior (*i.e*., walking, running, burrowing, inactive, and grooming). Preference for substrate type was also analyzed using another GLM. To compare body temperature of carabids that were prevented from burrowing into the soil with the addition of a nylon mesh to carabids allowed to engage in burrowing behavior, as well as interspecific differences, an LM was performed. The significance of each model term was assessed by a likelihood‐ratio test (LRT), the distribution of which, as usual, was considered as Chi‐square. A multiple comparison using the post hoc Tukey test was then performed using the emmeans package (Lenth, 2021) to evaluate differences among the 3 treatments for body temperature.

## Results

### Carabid cold tolerance

A total of 31 species were collected during the autumn sampling seasons and 38 during the spring sampling seasons (see Table S1). Of all the species collected, 10 species were present across the 3 landscape types (with at least 10 individuals for each type: simple [S.], intermediate [I.], and complex [C.]): *A. muelleri* (*n* = 311// S. = 119, I. = 99, C. = 93), *A. aenea* (*n* = 45// S. = 10, I. = 16, C. = 19), *A. dorsalis* (*n* = 126// S. = 58, I. = 46, C. = 22), *B. lampros* (*n* = 294// S. = 143, I. = 88, C. = 63), *B. quadrimaculatum* (*n* = 81// S. = 16, I. = 26, C. = 39), *B. tetracolum* (*n* = 84// S. = 14, I. = 45, C. = 25), *H. rufipes* (*n* = 172// S. = 58, I. = 75, C. = 39), *P. cupreus* (*n* = 249// S. = 95, I. = 53, C. = 101), *P. melanarius* (*n* = 233// S. = 99, I. = 105, C. = 29), and *T. quadristriatus* (*n* = 100// S. = 30, I. = 39, C. = 31). This represented a total of 1 695 carabids out of the 1 927 sampled (642 in the simple landscape, 592 in the intermediate landscape, and 461 in the complex landscape).

**Fig. 2:**
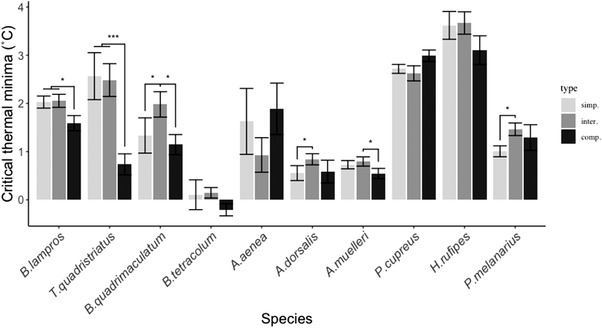
Carabid cold tolerance as measured by CT_min_ (±SE) sampled in the 3 landscape types: complex (comp), intermediate (inter), and simple (simp) considering all sampling seasons. Species are ordered by size, from smallest to largest. Each pairwise comparison between group level was calculated using a log‐rank test using the pairwise_survdiff function in R. Asterisks indicate significant *P* values: *P ≤ 0.05, **P ≤ 0.01, ***P ≤ 0.001.

Sampling period had no effect on carabid cold tolerance as measured by CT_min_ (*χ* ^2^ = 2.20, *df* = 1, *P* = 0.14). There was a significant effect of landscape type (simple, intermediate, complex) on the onset of carabid CT_min_ and significant inter‐species differences in the overall model. However, these effects were not interpretable as a high interaction effect was observed between landscape type and species (*χ* ^2^ = 127.09, *df* = 31, *P* < 0.001), suggesting that species differ in their sensitivity to landscape intensification. Carabid cold tolerance as measured by CT_min_ differed between landscape type for 6 of the 10 species considered in the model, based on pairwise comparisons (Benjamini–Hochberg adjustment). No effect of landscape was observed for *A. aenea*, *B. tetracolum*, *H. rufipes*, and *P. cupreus* (Fig. 2). For 3 species, the temperature of CT_min_ was significantly lower in complex landscapes compared to the 2 other landscape types (*B. lampros* and *T. quadristriatus*) or to the intermediate landscape type only (*A. muelleri*) (Fig. 2). This effect was most pronounced for *T. quadristriatus* for which individuals from complex landscapes (CT_min_ of 0.73 ± 0.22°C) maintained spontaneous movement to temperatures approximately 1.8°C lower than individuals from intermediate and simple landscapes (CT_min_ of 2.48 ± 0.34°C and CT_min_ of 2.56 ± 0.49°C, respectively) (Fig. 2). For 3 species, *A. dorsalis*, *B. quadrimaculatum*, and *P. melanarius*, values of CT_min_ obtained for individuals from the intermediate landscape were almost double what were obtained for individuals in the simple landscape (Fig. 2).

*P. cupreus* (CT_min_ of 2.79 ± 0.07°C) and *H. rufipes* (CT_min_ of 3.53 ± 0.16°C) showed the highest mean values of CT_min_ of the tested species. *P. cupreus* and *H. rufipes* were thus rendered immobile at significantly warmer temperatures than the other species. No significant difference was observed between *B. lampros* (CT_min_ of 1.94 ± 0.08°C), *T. quadristriatus* (CT_min_ of 1.97 ± 0.22°C), *B. quadrimaculatum* (CT_min_ of 1.43 ± 0.15°C), *P. melanarius* (CT_min_ of 1.25 ± 0.08°C), and *A. aenea* (CT_min_ of 1.48 ± 0.30°C), although these species showed an increased cold tolerance compared to *P. cupreus* and *H. rufipes*. The 3 remaining species displayed an increased cold tolerance: *A. dorsalis* (CT_min_ of 0.66 ± 0.09°C), *A. muelleri* (CT_min_ of 0.69 ± 0.05°C), and *B. tetracolum* (CT_min_ of 0.03 ± 0.09°C). The CT_min_ of *B. tetracolum* was significantly lower than the 2 other species.

Sampling period had a significant effect on carabid cold tolerance as measured by chill coma (the temperature at which the insect enters a cold‐induced torpor) (*χ* ^2^ = 4.54, *df* = 1, *P* = 0.03) with the mean temperature of chill coma for carabids sampled in autumn/winter (−5.81 ± 0.04°C) being significantly lower than the chill coma for carabids sampled in spring and summer (−5.70 ± 0.08°C). Landscape type had no significant effect on carabid cold tolerance as measured by chill coma (*χ* ^2^ = 2.28, *df* = 2, *P* = 0.32). An interaction effect was also observed between landscape type and species (*χ* ^2^ = 32.30, *df* = 12.37, *P* = 0.001) although with not enough variance between modalities to be detected in pairwise tests. A negative beta coefficient was obtained for *P. cupreus* only (−5.96 ± 0.07°C) when compared to *H. rufipes* as a baseline (−5.75 ± 0.08°C). For *P. melanarius*, the temperature of chill coma (−5.61 ± 0.06°C) was not significantly different from *H. rufipes*.

Carabid size was determined as follows: *A. aenea* 6.25 ± 0.23 mm (*n* = 35); *A. muelleri* 7.52 ± 0.08 mm (*n* = 273); *A. dorsalis* 6.28 ± 0.12 mm (*n* = 113); *B. lampros* 2.72 ± 0.03 mm (*n* = 194); *B. tetracolum* 5.14 ± 0.11 mm (*n* = 73); *H. rufipes* 12.50 ± 0.32 mm (*n* = 25); *P. cupreus* 9.74 ± 0.19 mm (*n* = 102); *P. melanarius* 14.98 ± 1.11 mm (*n* = 5); *T. quadristriatus* 2.93 ± 0.10 mm (*n* = 2). There was a significant effect of carabid size on cold tolerance (*χ* ^2^ = 48.39, *df* = 1, *P* < 0.001) with carabid size being positively correlated with the temperature of CT_min_ (positive coefficients *βi*). A reduced effect of landscape on the thermotolerance of larger carabids was observed. The larger species were either not affected by landscape type (*P. cupreus* and *H. rufipes)* or only in intermediate landscapes compared to simple landscapes (*P. melanarius*) (Fig. 2). Two of the 3 species for which cold tolerance was affected by complex landscape were among the smallest species sampled (*T. quadristriatus* and *B. lampros)* (Fig. 2).

### Carabid behavioral thermoregulation

The activity level, as determined by exhibited walking behavior, significantly differed between the 3 carabid species (*χ* ^2^ = 22.36, *df* = 2, *P* < 0.001), and between the treatment group (cold stress *vs*. temperature control) (*χ* ^2^ = 6.01, *df* = 1, *P* = 0.01). Here, *P. cupreus* was shown to be 3 times more active than *H. rufipes* (Tukey, *P *< 0.001), engaging in more frequent walking behavior while other inter‐species differences were not significant (Tukey, *P* > 0.05). Cold stressed carabids significantly engaged in less frequent walking behavior than carabids in the control group (Tukey, *P* = 0.01).

Very little grooming or running behavior or periods of inactivity were observed during the duration of the experiment regardless of the temperature treatment applied. When exposed to a cold stress, carabid beetles did not display different patterns of grooming (*χ* ^2^ = 0.07, *df* = 2, *P* = 0.79), running (*χ* ^2^ = 0.41, *df* = 2, *P* = 0.52), or inactivity (*χ* ^2^ = 1.28, *df* = 2, *P* = 0.25) compared to the control group. No inter‐specific differences were measured for grooming (*χ* ^2^ = 1.88, *df* = 2, *P* = 0.39) and running (*χ* ^2^ = 3.29, *df* = 2, *P* = 0.19) but *P. melanarius* was significantly more inactive than the other 2 species (*χ* ^2^ = 24.39, *df* = 2, *P *< 0.001).

An interspecific preference for substrate type was also suggested for 3 of the substrate types: wheat (*χ* ^2^ = 41.86, *df* = 2, *P *< 0.001), bare soil without shelter (*χ* ^2^ = 15.43, *df* = 2, *P *< 0.001), and bare soil with shelter (*χ* ^2^ = 80.83, *df* = 2, *P *< 0.001), but not for the substrate with drilled holes (*χ* ^2^ = 3.74, *df* = 2, *P* = 0.15). *P. melanarius* was observed to spend more time in the substrate containing bare soil and an artificial shelter than either *P. cupreus* or *H. rufipes* (*P *< 0.001, Tukey) and at least 4 times less in the substrate containing wheat than the 2 other species (*P *< 0.001, Tukey). *P*. *cupreus* spent significantly more time in substrate containing bare soil without shelter than the 2 others species (Tukey, *P *< 0.01).

Of the different behaviors investigated, burrowing behavior appeared to be the behavior most commonly employed by carabid beetles when exposed to a cold stress, with carabids from the cold stress treatment group spending significantly more time buried in the substrate (19.35 ± 1.30 min) than carabids in the control group (14.60 ± 2.76 min) (*χ* ^2^ = 8.37, *df* = 1, *P* = 0.003). Burrowing time also significantly differed between the 3 species (*χ* ^2^ = 16.64, *df* = 2, *P *< 0.001), with *H. rufipes* remaining buried in the substrate for a longer period of time (21.06 ± 1.44 min) than *P. cupreus* (17.06 ± 1.76 min) (Tukey, *P* = 0.02) and *P. melanarius* (13.00 ± 4.46 min) (Tukey, *P *< 0.001). Cold stressed *P. cupreus*, the carabid displaying the lowest temperature of chill coma, burrowed into the soil later and thus at colder temperatures (14.25 ± 3.06 min) than cold stressed *H. rufipes* (7.37 ± 2.01 min) (Tukey, *P *< 0.001) and cold stressed *P. melanarius* (4.40 ± 1.60 min) (Tukey, *P *< 0.001). Burial latency between *P. melanarius* and *H. rufipes* were not significantly different (Tukey, *P* = 0.11).

Body temperature was found to significantly differ between the treatment groups (*χ* ^2^ = 133.76, *df* = 2, *P *< 0.001), although did not differ between the 3 species (*χ* ^2^ = 0.11, *df* = 2, *P* = 0.89). When carabids were prevented from burrowing into the soil with the addition of a nylon mesh (the behavioral control), recorded body temperature (5.78 ± 0.29°C) was significantly lower than the body temperature of carabids allowed to engage in burrowing behavior (8.13 ± 0.29°C) (Tukey, *P *< 0.001). In addition, the body temperature of the temperature control group (14.91 ± 0.30°C) was significantly higher than the body temperature of carabids in the cold stress group and the behavioral control group (Tukey, *P *< 0.001).

## Discussion

The current study revealed a landscape composition effect on the cold tolerance of carabid beetles, although species differed in their sensitivity to landscape composition, thus partly confirming hypothesis 1. Season did not influence the landscape effect on carabid cold tolerance, refuting hypothesis 2. Hypothesis 3, which predicted a reduced effect of landscape complexity on the thermotolerance of larger‐bodied species, was partially supported since there was no effect of landscape on the CT_min_ of *H. rufipes* and *P. cupreus* or the chill coma temperature of *H. rufipes*, *P. cupreus* and *P. melanarius*; the 3 largest species of carabid in the current study. In support of Hypothesis 4, which predicted that carabid beetles would display behavioral thermoregulation to escape unfavorable cold temperatures, burrowing behavior was found to be the primary behavioral response to cold stress, acting to significantly alter carabid body temperature. Furthermore, *P. cupreus*, the carabid possessing the lowest chill coma temperature, burrowed into the soil at lower temperatures (i.e., remained exposed for longer durations) than *H. rufipes* and *P. melanarius*, offering support to Hypothesis 5 that more cold tolerant carabids will be less reliant on behavioral thermoregulation.

### Landscape effects on cold tolerance

Landscape components such as topography, vegetation type, and cover impact the microclimate of the landscape (Chen *et al*., 1999). Indeed, complex agricultural landscapes have been shown to be significantly colder, but less variable, than simple landscapes (Tougeron *et al*., 2016; Alford *et al*., 2018). This is owing, in part, to the windbreak function of trees and hedgerows, resulting in a reduction in wind speed and subsequent retention of denser, cooler air (Quénol & Beltrando, 2006). Furthermore, as a landscape becomes increasingly closed, it is exposed to less short radiation from the sun which would otherwise raise the local temperature (Chen *et al*., 1999; Suggitt *et al*., 2011). Due to the colder mean temperatures experienced in complex landscapes, it was hypothesized that carabids originating from complex landscapes would be more cold tolerant than carabids originating from intermediate and simple landscapes (hypothesis 1). This hypothesis was supported by results on CT_min_ obtained for *A. muelleri*, *B. lampros*, and *T. quadristriatus*. However, for *A. dorsalis*, *B. quadrimaculatum*, and *P. melanarius*, individuals were more cold tolerant when originating from both complex and simple landscapes, although this difference was only significant for *A. dorsalis* and *P. melanarius* between simple and intermediate landscapes. When exposed to cold temperatures, a number of factors influence insect thermotolerance and survival including temperature extremes, cooling rate, exposure duration, and the extent to which temperature fluctuates around freezing (Sinclair *et al*., 2003; Terblanche *et al*., 2007). The importance of temperature extremes in dictating ectotherm thermal resistance is commonly accepted (Bahrndorff *et al*., 2006; Paaijmans *et al*., 2013; Estay *et al*., 2014), although increasing research is suggesting that insects may differ in their sensitivity to aspects of the thermal environment and alter their physiological thermotolerance accordingly. Indeed, recent research has indicated that increased temperature variation increases the cold tolerance of the fruit fly *Drosophila melanogaster* (Diptera: Drosophilidae) and the moth *Helicoverpa armigera* (Lepidoptera: Noctuidae), whilst reducing the cold tolerance of the aphid *Acyrthosiphon pisum* (Hemiptera: Aphididae) (Estay *et al*., 2014), indicating that whilst temperature extremes are important cues to the fruit fly and the moth, it is constant mean temperatures that are the more salient cue in aphid thermotolerance. Likewise, in a field study in temperate agricultural landscapes, the thermotolerance of *Aphidius* wasps responded to temperature fluctuations (Tougeron *et al*., 2016), whilst the thermotolerance of cereal aphids responded to temperature means (Alford *et al*., 2018). It is possible that carabid beetles may also differ in their sensitivity to temperature means and extremes, offering explanation as to why enhanced thermotolerance was observed in carabids originating from complex and simple landscapes.

Interestingly, the observed landscape effect on carabid thermotolerance (as determined by CT_min_) was not influenced by season, refuting hypothesis 2 which stated that landscape effects on carabid thermotolerance would be more pronounced in autumn and less pronounced or absent in spring. In temperate environments, environmental cues such as declining temperatures and shortening day lengths trigger seasonal cold adaptation in insects, enabling tolerance to the cold temperatures experienced during winter months (Teets & Denlinger, 2013). On the onset of spring, mean temperatures become warmer and less extreme. Any acquired thermotolerance, which is costly to maintain (Block, 1991; Voituron *et al*., 2002; Schou *et al*., 2015), should be lost on cessation of winter temperatures, and any effect of landscape on thermotolerance should be reduced or lacking. This has previously been reported for parasitoid wasps (Tougeron *et al*., 2016) and cereal aphids (Alford *et al*., 2018), whereby landscape effects on thermotolerance were significantly reduced in spring months compared to winter months. This was not observed for carabids in the current study, with season having no effect on carabid CT_min_ along a landscape intensification gradient. Since carabids were sampled commencing September and March for an autumn and spring population, respectively, it is possible that sampling did not capture peak seasonal cold acclimation in carabids, thereby preventing a seasonal effect from being elucidated.

Whilst an effect of landscape on the cold tolerance of *A. muelleri*, *A. dorsalis*, *B. lampros*, *B. quadrimaculatum*, *P. melanarius*, and *T. quadristriatus* was reported, there was no effect of landscape on the CT_min_ of *A. aenea*, *B. tetracolum*, *H. rufipes*, and *P. cupreus*, nor the chill coma temperature of *H. rufipes*, *P. cupreus*, and *P. melanarius*. This finding partially supports hypothesis 3 which stated that there would be a reduced effect of landscape on the thermotolerance of larger carabids, since *H. rufipes*, *P. cupreus*, and *P. melanarius* were the 3 largest species collected in the study area. *A. aenea* and *B. tetracolum* were intermediate sized carabids. Any increased ability to move throughout the landscape may hinder local acclimation to microclimatic variation along a landscape intensification gradient. In a tracking study conducted in cereal fields of Uppsala, Sweden, it was found that *P. melanarius* traveled average distances of 5.3 m, and *H. rufipes* average distances of 7.3 m, during their main activity period, although distances of up to 44.0 m were reported for *P. melanarius* (Wallin & Ekbom, 1988). At least for the largest 2 carabids in the current study, comparatively large‐scale movements throughout the landscape are thus known and, as such, may prevent patterns in physiological thermotolerance at the landscape scale. Indeed, enhanced mobility of larger insects preventing local patterns in thermotolerance has recently been reported in bees. In a study into the critical thermal maximum (CT_max_) of the bees *Agapostemon sericeus* (Hymenoptera: Halictidae), *Apis mellifera* (Hymenoptera: Apidae) and *Bombus impatiens* (Hymenoptera: Apidae) along an urbanization gradient, Burdine and McCluney (2019) found no difference in thermotolerance between urban and rural sites. This led authors to conclude that greater mobility may prevent local acclimation and adaptation in the study bees. Furthermore, any local adaptation that did occur could be erased by genetic mixing.

### Carabid behavioral thermoregulation

Burrowing behavior was revealed to be the primary behavioral response exhibited by *H. rufipes*, *P. cupreus*, and *P. melanarius* when faced with a cold stress. Indeed, soil represents a more stable thermal environment than air, with thermal stability increasing with soil depth (Scharringa, 1976; Illston & Fiebrich, 2017) and vegetation cover further acting to reduce temperature variation (Scharringa, 1976; Oliver *et al*., 1987). In the current study, engaging in burrowing behavior enabled carabids to maintain body temperature approximately 2°C higher than carabids prevented from engaging in burrowing behavior, thus supporting hypothesis 4 which stated that carabid beetles will display behavioral thermoregulation to escape unfavorable cold temperatures. Burrowing behavior therefore provides thermoregulatory benefits to carabids during a cold stress. Indeed, behavioral thermoregulation more generally enables species to deal with variable environments. Nonetheless, for many species, behavioral thermoregulation is considered an unavailable or insufficient mechanism, with small body size considered a primary factor in limiting the ability of organisms to regulate body temperature (Gilchrist, 1995). Indeed, Merrick and Smith (2004) suggest that beetles below 2 g in weight are incapable of maintaining thoracic temperature independently of ambient temperature, most likely the consequence of a larger surface area to volume ratio (Merrick & Smith, 2004). Authors further state that, for smaller beetles less than 1 g in weight, wing loading, and wing beat frequency may play an important role in the maintenance of body temperature (Merrick & Smith, 2004). In the current study, *P. melanarius* represents one of the larger of the carabid species studied, weighing approximately 160 mg (Jonsson *et al*., 2018); far below the 2 g threshold required for maintenance of body temperature as suggested by Merrick and Smith (2004). However, the current study revealed that burrowing behavior can significantly raise the thoracic temperature of carabids by approximately 2°C, providing evidence that behavioral thermoregulation is a valuable method of body temperature regulation in beetles weighing 160 mg and below.

With burrowing behavior established as an important method of behavioral thermoregulation in carabid beetles, it was further hypothesized (hypothesis 5) that more cold tolerant species will be less reliant on behavioral thermoregulation, and instead utilize their inherent physiological thermotolerance to withstand a cold stress. Tentatively supporting this hypothesis was the finding that the species with the highest temperature of chill coma, *H. rufipes*, remained buried in the substrate for longer periods of time than the species with the lowest temperature of chill coma, *P. cupreus*. In a previous study investigating beetle overwintering in Japan, it was found that more accomplished diggers could avoid cold temperatures by burrowing deeper into the ground, thereby negating the need to develop frost resistance (Hoshikawa *et al*., 1988). Since incurring seasonal cold acclimation is costly (Block, 1991; Voituron *et al*., 2002; Schou *et al*., 2015), it is possible that burrowing behavior results in a reduced investment in physiological mechanisms of cold tolerance, suggesting that a trade‐off between physiological thermotolerance and behavioral thermoregulation may exist. A reduced cold tolerance would thus be the consequence of burrowing behavior, and not instead a prerequisite to engage in burrowing behavior.

## Conclusion

A clear effect of landscape on the thermotolerance of carabid beetles was observed in the current study. However, carabid beetles did not respond in a similar manner to thermal conditions and changes in microhabitat and microclimate. The lack of landscape effect on the thermotolerance of larger species suggests that increased mobility may hinder local acclimation to thermal conditions. Furthermore, burrowing behavior, although revealed to be an important method of carabid behavioral thermoregulation, may further act to hinder local acclimation. If behavioral thermoregulation impedes an acclimatory response in carabid thermotolerance along a microclimatic gradient, it is likely that physiological mechanisms involved in thermotolerance are not under climate‐induced selection pressures, with implications for the long‐term evolutionary potential of carabid thermotolerance.

## Disclosure

The authors declare that they have no conflict of interest.

## Supporting information


**Table S1** Carabid beetle species and associated *n* numbers collected during (a) autumn 2013 and 2014 and (b) spring 2014 and 2015.

**Appendix 1** evaluation and comparison of the mean CT_min_ of each species of carabids.
