# Supplementary material for: Landscape effects on the thermotolerance of carabid beetles and the role of behavioral thermoregulation
Source: Insect Sci. 2022 May 16;30(1):251–63. doi: 10.1111/1744-7917.13044 (PMC10084217; doi:10.1111/1744-7917.13044)
Supplement: Supplementary file 1 — Table S1 Carabid beetle species and associated n numbers collected during (a) autumn 2013 and 2014 and (b) spring 2014 and 2015. [file INS-30-251-s001.docx]

**Table S1** Carabid beetle species and associated *n* numbers collected during a) autumn 2013 and 2014 and b) spring 2014 and 2015. Species names marked with an asterisk denote species found in both autumn and spring samples.

a)

| **Carabid species** | ***n* A2013** | ***n* A2014** |
| --- | --- | --- |
| *Abax parallelepipedus* | 2 | 4 |
| *Agonum muelleri** | 3 | 5 |
| *Agonum nigrum* | 0 | 1 |
| *Amara aenea** | 1 | 2 |
| *Amara communis** | 0 | 1 |
| *Amara convexior** | 0 | 1 |
| *Amara similata** | 0 | 4 |
| *Anchomenus dorsalis** | 0 | 2 |
| *Anisodactylus binotatus** | 1 | 0 |
| *Asaphidion flavipes** | 1 | 0 |
| *Bembidion lampros** | 118 | 74 |
| *Bembidion quadrimaculatum** | 30 | 48 |
| *Bembidion tetracolum** | 2 | 0 |
| *Calathus melanocephalus* | 1 | 0 |
| *Diachromus germanus* | 0 | 2 |
| *Harpalus affinis** | 0 | 3 |
| *Harpalus attenuatus* | 0 | 1 |
| *Harpalus rufipes** | 88 | 58 |
| *Laemostenus terricola* | 2 | 0 |
| *Loricera pilicornis** | 0 | 1 |
| *Nebria brevicollis** | 5 | 0 |
| *Notiophilus quadripunctatus** | 0 | 1 |
| *Ophonus puncticeps* | 0 | 1 |
| *Oxypselaphus obscurus* | 1 | 0 |
| *Platynus livens* | 0 | 2 |
| *Poecilus cupreus** | 7 | 67 |
| *Pterostichus madidus* | 15 | 45 |
| *Pterostichus melanarius** | 136 | 91 |
| *Pterostichus strenuus* | 0 | 1 |
| *Pterostichus vernalis** | 0 | 1 |
| *Trechus quadristriatus** | 46 | 51 |

b)

| **Carabid species** | ***n* S2014** | ***n* S2015** |
| --- | --- | --- |
| *Agonum afrum* | 0 | 1 |
| *Agonum muelleri** | 94 | 209 |
| *Agonum sexpunctatum* | 0 | 5 |
| *Amara aenea** | 29 | 13 |
| *Amara communis** | 2 | 0 |
| *Amara convexior** | 1 | 0 |
| *Amara familiaris* | 6 | 0 |
| *Amara montivaga* | 3 | 0 |
| *Amara ovata* | 1 | 0 |
| *Amara similata** | 6 | 0 |
| *Anchomenus dorsalis** | 56 | 67 |
| *Anisodactylus binotatus** | 5 | 0 |
| *Asaphidion flavipes** | 10 | 1 |
| *Bembidion iricolor* | 11 | 1 |
| *Bembidion lampros** | 71 | 31 |
| *Bembidion mannerheimii* | 0 | 1 |
| *Bembidion obtusum* | 3 | 1 |
| *Bembidion properans* | 4 | 0 |
| *Bembidion quadrimaculatum** | 3 | 0 |
| *Bembidion tetracolum** | 29 | 53 |
| *Chlaenius nigricornis* | 1 | 0 |
| *Harpalus affinis** | 4 | 1 |
| *Harpalus latus* | 1 | 0 |
| *Harpalus rubripes* | 3 | 0 |
| *Harpalus rufipes** | 2 | 24 |
| *Harpalus signaticornis* | 1 | 0 |
| *Loricera pilicornis** | 17 | 3 |
| *Nebria brevicollis** | 2 | 0 |
| *Nebria salina* | 20 | 0 |
| *Notiophilus biguttatus* | 2 | 0 |
| *Notiophilus quadripunctatus** | 7 | 1 |
| *Poecilus cupreus** | 92 | 83 |
| *Pterostichus melanarius** | 1 | 5 |
| *Pterostichus niger* | 1 | 0 |
| *Pterostichus vernalis** | 4 | 0 |
| *Stenolophus teutonus* | 1 | 0 |
| *Trechus quadristriatus** | 3 | 0 |
| *Trechus rubens* | 0 | 5 |
