# Supplementary material for: Landscape effects on the thermotolerance of carabid beetles and the role of behavioral thermoregulation
Source: Insect Sci. 2022 May 16;30(1):251–63. doi: 10.1111/1744-7917.13044 (PMC10084217; doi:10.1111/1744-7917.13044)
Supplement: Supplementary file 2 — Appendix 1 evaluation and comparison of the mean CTmin of each species of carabids. [file INS-30-251-s002.docx]

Appendix 1: evaluation and comparison of the mean CT_min_ of each species of carabids.

The sample of CT_mins_ of each carabid species appear clustered by fields (random effect) and stratified by the landscape types. We have three strata: complex, intermediate and simple. Let us, considering a given species, design by $i$the index of the stratum, by $j$the index of the field and by $k$ the index of the individual.

The CT_min_ of the $k^{th}$ individual of the species is observed in the $j^{th}$ field of the $i^{th}$ stratum and the average CT_min_ of that species is calculated :

As the variable of interest is an individual quantitative characteristic, each field, as a cluster, has the weight $w_{ij}=\frac{n_{ij}}{n_{i}}$, and all the individuals of the cluster share a common random variable $A_{ij}$, that we suppose normal, of null expectation and of variance $\sigma_{A}^{2}$ (the between cluster component of the variance).

Inside the stratum $i$, the correct estimation of the mean is the weighted mean of the different clusters:

$$CT_{i..}=\sum_{j=1}^{n_{j}} w_{ij}CT_{ij.}$$

As we suppose that the number of trapped individuals is proportional to the abundance of the species in the given field, formula is equal to the ordinary mean within the stratum, but its variance is different from that of the ordinary (non-clustered) mean because of the intra-cluster correlation caused by the common realisation of the random variable $A_{ij}$.

The statistical model of the individual value is:

$$CT_{ijk}=\mu+\alpha_{i}+A_{ij}+\varepsilon_{ijk}$$

Where $\mu$is the overall mean of the population, $\alpha_{i}$the fixed effect of the stratum $i$, $A_{ij}$ the random variable attached to cluster $i$of variance $\sigma_{A}^{2}$, as defined above, and $\varepsilon_{ijk}$the residual attached to each individual, a random variable of null expectation and $\sigma_{r}^{2}$variance (residual variance).

The variance of the mean inside a cluster (field) is :

$$\frac{1}{n_{ij}}\left(n_{ij}\sigma_{A}^{2}+\sigma_{r}^{2}\right)=\sigma_{A}^{2}+\frac{\sigma_{r}^{2}}{n_{ij}}$$

And the estimates of $\sigma_{A}^{2}$ and $\sigma_{r}^{2}$can be obtained by the method of moments from a fixed analysis of variance among clusters:

$$\hat{\sigma}_{A}^{2}=\frac{MS_{A}-MS_{R}}{\bar{n}_{i}}$$

Where $MS_{A}$is the between clusters mean square, $MS_{R}$ the residual mean square and $\bar{n}_{i}$the average number of carabs among the clusters.

Thus, the estimated variance of the mean in the whole stratum is:

$$\mathrm{var}\left(CT_{i..}\right)=\sum_{j=1}^{n_{j}} w_{ij}^{2}\mathrm{var}\left(CT_{ij.}\right)=\sum_{j=1}^{n_{j}} w_{ij}^{2}\left(\hat{\sigma}_{A}^{2}+\frac{\hat{\sigma}_{r}^{2}}{n_{ij}}\right)$$

The weights of the stratum $i$is $\frac{n_{i.}}{N}$ and thus, the variance of the overall mean is :

$$\mathrm{var}\left(CT_{...}\right)=\sum_{i=1}^{m} \frac{n_{i}^{2}}{N^{2}}\sum_{j=1}^{n_{j}} \frac{n_{ij}^{2}}{n_{i}^{2}}\left(\sigma_{A}^{2}+\frac{\sigma_{r}^{2}}{n_{ij}}\right)$$

which is the Standard error of the estimated mean of the species.

and the overall stratified mean:

$$CT_{...}=\sum_{i=1}^{m} w_{i}CT_{i.}$$

It is therefore straightforward to calculate the mean and standard error of each species and to compare all of them two by two with a Bonferroni procedure.

This procedure assumes a normal distribution of the CT_min_, which seems contradictory with the analysis performed using the Cox model. However, the Cox model does not provide an easy procedure to compare CT_min_ means. As the number of individuals per cluster is fairly large, we are protected by the central limit theorem, and moreover by the fast convergence of variances to the Chi-square distributions. Furthermore, the estimations of $\sigma_{A}^{2}$ and $\sigma_{r}^{2}$do not depend on the normality hypothesis, and Bonferroni is used as an exceedingly conservative procedure.
